# Molecular basis of natural tolerance to glyphosate in *Convolvulus arvensis*

**DOI:** 10.1038/s41598-019-44583-8

**Published:** 2019-05-31

**Authors:** Zhaofeng Huang, Yan Liu, Chaoxian Zhang, Cuilan Jiang, Hongjuan Huang, Shouhui Wei

**Affiliations:** 1 Key Laboratory of Weed Science, Institute of Plant Protection (IPP), Chinese Academy of Agricultural Sciences (CAAS), Beijing, 100193 China; 2 Institute of Environment and Plant Protection, Chinese Academy of Tropical Agricultural Sciences, Haikou, 570100 China

**Keywords:** Abiotic, Molecular biology, Plant molecular biology

## Abstract

*Convolvulus arvensis* is a troublesome weed that is naturally tolerant to glyphosate. This weed tolerates glyphosate at a rate 5.1 times higher than that of glyphosate-susceptible *Calystegia hederacea*. Glyphosate-treated *C*. *arvensis* plants accumulated less shikimic acid than *C*. *hederacea* plants. The overexpression of *EPSPS* genes from the two species in transgenic *Arabidopsis thaliana* resulted in similar glyphosate tolerance levels. qPCR of genomic DNA revealed that the *EPSPS* copy number in *C*. *arvensis* was approximately 2 times higher than that in *C*. *hederacea*. Moreover, glyphosate treatment caused a marked increase in *EPSPS* mRNA in *C*. *arvensis* compared to *C*. *hederacea*. GUS activity analysis showed that the promoter of *CaEPSPS* (*CaEPSPS-P*) highly improved GUS expression after glyphosate treatment, while no obvious differential GUS expression was observed in *ChEPSPS-P* transgenic *A*. *thaliana* in the presence or absence of glyphosate. Based on the obtained results, two coexisting mechanisms may explain the natural glyphosate tolerance in *C*. *arvensis*: (i) high *EPSPS* copy number and (ii) specific promoter-mediated overexpression of *EPSPS* after glyphosate treatment.

## Introduction

Glyphosate is a nonselective, foliar-applied herbicide that has been used to manage annual, perennial, and biennial herbaceous species of grasses, sedges, and broadleaf weeds^1,2^. It affects aromatic amino acid biosynthesis by inhibiting 5-enolpyruvyl-shikimate-3-phosphate synthase (EPSPS), a nuclear-encoded, plastid-localized enzyme in the shikimate pathway^3^. Glyphosate has become the most widely used herbicide in the world due to its advantage of broad-spectrum, low toxicity, and low soil residual activity^4^. However, the widespread and intensive use of glyphosate over years imposes selective pressure on weeds^5,6^. Since glyphosate resistance was first found in rigid ryegrass (*Lolium rigidum*)^7^ in Australia in 1996, 43 weed species with resistance to glyphosate have been detected^8^.

Mechanisms of glyphosate resistance are classified as target-site and non-target site. Target-site resistance is caused by mutations in EPSPS that decrease its binding affinity for glyphosate, or by EPSPS overexpression, which allows the plant to produce adequate EPSPS to maintain the synthesis of aromatic amino acids. Single amino acid substitutions in EPSPS at position 106 from proline to serine (P106S), alanine (P106A), threonine (P106T), or leucine (P106L) have been identified in *Eleusine indica*^9^, *L*. *rigidum*^10,11^, *Lolium multiflorum*^12^, *Echinochloa colona*^13^, and *Amaranthus tuberculatus*^14,15^. Additionally, a double amino acid substitution (T102I + P106S) in *E*. *indica*^16,17^ and *Bidens Pilosa*^18^ in certain populations was found and reported to confer a higher glyphosate resistance level than that conferred by the single P106S mutation.

*EPSPS* overexpression through increased *EPSPS* copy number confers glyphosate resistance in *A*. *palmeri*^19^, *L*. *multiflorum*^20^, *A*. *spinosus*^21^, and *A*. *tuberculatus*^22,23^. For glyphosate-resistant *A*. *palmeri*, increased *EPSPS* copy number produces abundant enzymes to maintain the shikimate pathway^18^. Furthermore, *EPSPS* overexpression through elevated *EPSPS* transcript levels after glyphosate treatment is associated with glyphosate tolerance in *Dicliptera chinensis*^24^ and *Ophiopogon japonicus*^25^.

Reduced glyphosate absorption, translocation^26,27^, and vacuolar sequestration^28^ are the main non-target glyphosate resistance mechanism. To protect the young meristematic tissue, resistant plants sequester glyphosate within the vacuoles of the leaves^29,30^. Maintaining glyphosate in vacuolar tissues by ABC transporters to avoid damage was identified to be responsible for glyphosate resistance^31,32^. Furthermore, studies have reported that chloroplast proteins played an important role in glyphosate resistance in *Conyza canadensis*^33^.

Field bindweed (*Convolvulus arvensis* L) is a perennial weed in the morning-glory family. It is considered one of the most troublesome weeds threatening wheat and cotton production in China^34^. *C*. *arvensis* was the first weed reported to be naturally tolerant to glyphosate^35^. Previous studies aimed at illuminating the glyphosate tolerance mechanism in *C*. *arvensis* have mainly focused on glyphosate absorption and translocation. However, there were no obvious differences in absorption and translocation^36,37^. Until recently, the tolerance mechanism has not been fully understood. As *C*. *arvensis* is naturally tolerant to glyphosate, and a susceptible population in China was not obtained in our previous studies. Therefore, glyphosate-susceptible *Calystegia hederacea* was used as a control because *C*. *hederacea* belongs to the Convolvulaceae family and shares similar biological characteristics with *C*. *arvensis* in many aspects, such as perennial, vine climbing, and rapid growth^38^. In this article, we investigated the mechanism of glyphosate tolerance in *C*. *arvensis* with physiological (shikimic acid accumulation) and molecular (*EPSPS* cloning, overexpression of *EPSPS* gene, and *EPSPS* gene expression pattern) approaches. We cloned the *EPSPS* genes of *C*. *hederacea* and *C*. *arvensis* and inserted the *EPSPS* gene into the common model plant *Arabidopsis thaliana*, which is an excellent tool for research in plant biology^39^. We examined the glyphosate tolerance of *EPSPS-*transgenic *A*. *thaliana*. We also compared the basal and glyphosate-induced mRNA levels of *EPSPS* from the two species.

## Materials and Methods

### Plant material and growth conditions

Seeds of *C*. *arvensis* and *C*. *hederacea* collected in Beijing, China were germinated in Petri dishes with moist filter paper in an illumination incubator (25 °C day/night temperature). Individual seedlings in the cotyledon growth stage were transplanted into pots (5 cm radius; 6 seedlings per pot) containing a 1:1 (*V ⁄ V*) peat: sand sterile potting mix. The plants were placed in a greenhouse with an average day/night temperature of 25/20 °C and a 12-h photoperiod under artificial illumination (300 μmol m^−2^ s^−1^). The plants were watered as needed.

### Glyphosate dose–response assay

Plants at the 5–6 leaf stage were sprayed with glyphosate (Roundup Ultra, 41% glyphosate isopropylammonium, Monsanto, USA) at doses of 0, 250, 500, 1000, 2000, 4000 and 8000 g ha^−1^ using a research track sprayer (3WPSH-500D), which delivered 450 L ha^−1^ spray solution at 0.3 MPa. All treatments contained 3 replicate pots (6 plants per pot). Plants were assessed 14 days after treatment (DAT). All aboveground plant materials were cut and dried at 60 °C for 72 h. Dry weight was measured when constant weight was achieved. The experiment was arranged in a completely randomized design and was repeated two times with three replications each.

### Shikimate accumulation *in vivo* assay

Plants at the 5–6 leaf stage sprayed with 1000 g ha^−1^ glyphosate were harvested at 2, 4, 6, 8, 10 and 12 DAT, and foliar tissue samples were stored at −80 °C until further processed. Determination of shikimate accumulation in *C*. *hederacea* and *C*. *arvensis* tissue was conducted spectrophotometrically according to Chen^40^. Shikimic acid was detected using a double-beam spectrophotometer at 380 nm. The determination of the shikimic acid concentration was based on a shikimate (Sigma-Aldrich, Saint Louis, MO, USA). 99% purity) standard curve.

### *EPSPS* gene cloning and sequence analysis

Leaves of *C*. *hederacea* and *C*. *arvensis* were sampled and ground to fine powders in liquid nitrogen, and the total RNA was extracted with the RNAprep Pure Plant Kit (Tiangen Biotech Co., Ltd., China) following the manufacturer’s protocol. First-strand complementary DNA (cDNA) was amplified with random primers using EasyScript First-Strand cDNA Synthesis SuperMix (TransGen Biotech, China). The final cDNA was stored at −20 °C.

The primer pair EPSPS-cf and EPSPS-cr was designed from plant *EPSPS* gene sequences in NCBI. PCR was performed in a thermal cycler as follows: 5 min at 95 °C; 30 s at 95 °C; 30 s at 57 °C; 35 s at 72 °C (35 cycles); and 10 min at 72 °C. The amplified product was purified and cloned into the pMD19-T vector (Takara, Japan) for sequencing. The sequence obtained from the conserved region was used to design the 5′-end and the 3′-end primers. Fragments amplified by 5′ and 3′ RACE were purified, cloned into the pMD19-T vector and sequenced. Because of their high homology, ChEPSPS-f and ChEPSPS-r were designed to amplify the full-length *EPSPS* gene of *C*. *hederacea* according to that of *C*. *arvensis*. Sequence assembly and comparative analyses of the *EPSPS* genes from the two species were conducted using DNAMAN (Version 5.0).

The promoters of *EPSPS* from *C*. *hederacea* and *C*. *arvensis* were amplified with the gwEPS-1, gwEPS-2, and gwEPS-3 primers of the Universal Genome Walker^TM^ Kit (Clontech, USA) following the manufacturer’s protocol. The sequences of primers used in the present study are listed in Table 1. The prediction of *cis*-acting elements in the promoters was performed by using the software Plant-CARE.

**Table 1:** Primers used in this study.

Primer name	Primer sequence (5′ to 3′)	Purpose of the primers
EPSPS-cf	TGGTCTTAAGCAGCTTGGCGC	Amplify the core of *EPSPS*
EPSPS-cr	CACTGTTGCTCCCAACTTTCTT	
EPSPS-5	GCGCCAAGCTGCTTAAGACCA	5′ RACE
EPSPS-3	GCAGGAACAGCAATGCGTCC	3′ RACE
ChEPSPS-f	ATACCCACCAAATTCAATTAAGAGGT	Amplify the full length of *ChEPSPS*
ChEPSPS-r	ACCGGCTCAACCATTACAAGAAA	
gwEPS-1	CCTTCTACGGTTGCTCGCTGAATTGC	TAIL-PCR of the *EPSPS* promoter
gwEPS-2	TGAGAAAGGGCAGCAAGAAGGAGAA	
gwEPS-3	CACAATCTCCTCCGGTGCCATTGAC	
EPS-1f	TCTAGAATGGCGCAAGTGAACAACA	Amplify the full length of *EPSPS*
EPS-1r	CCCGGGTCAATGCTTGGAGAACTTG	
CaEPS-Pf	TAAACCTCTTAATTGAATTT	Amplify *CaEPSPS-P*
CaEPS-Pr	GGTATTTTAAAAGAGGCGTG	
ChEPS-Pf	GGACTCACTAGCTATCGCAG	Amplify *ChEPSPS-P*
ChEPS-Pr	GGTATTTTGAAAGAGGCGTG	
Q-EPS-f	GGTCCTTTCACCGTAACAC	qRT-PCR analysis of the *EPSPS* gene
Q-EPS-r	GGGGAGGTCAGAAATACA	
GAPDH-f	AACTGTCTTGCTCCTTTGGCTA	qRT-PCR analysis of the *GAPDH* gene
GAPDH-r	AGAACTTTCCCAACAGCCTTGGC	

### Quantitative PCR (qPCR) analysis

The relative *EPSPS* copy number was estimated using genomic DNA. Total DNA from young leaves (100 mg) of the two species from three plants of each replicate was extracted using the New Plant Genome Extraction Kit (Tiangen Biotech Co., Ltd., China). After eluting in double-distilled water, genomic DNA quality and concentration were determined spectrophotometrically, and the DNA samples were stored at −20 °C.

The *EPSPS* expression level was determined using mRNA extracted from plants after glyphosate treatments. Plants sprayed with 1000 g a.e. ha^−1^ glyphosate at the 5–6 leaf stage were harvested at 0.5, 1, 2, 4, 6 and 8 DAT. The leaves (the uppermost three leaves, 100 mg) of the two species were sampled from three plants of each replicate and ground to a fine powder in liquid nitrogen, and the total RNA was extracted by using the RNAprep Pure Plant Kit (Tiangen, China) following the manufacturer’s protocol. After elution of total RNA in double-distilled water, DNase I was added to digest any contaminating DNA and then removed. The cDNA was amplified with random primers using the EasyScript First-Strand cDNA Synthesis SuperMix (TransGen Biotech, China).

qPCR was performed in 96-well plates on the ABI 7500 real-time PCR system with the SYBR Green I Master Mix (Invitrogen, USA). To quantify the copy number and expression level of *EPSPS*, the housekeeping *GAPDH* gene was used as the internal control gene because the *GAPDH* gene did not vary across the samples based on our qPCR results (data not shown). The primer sequences used in this study are listed in Table 1. Melting curves were performed before each qPCR experiment to assess the specificity of the primers. The following two-step real-time PCR detection system was used: 15 s at 95 °C and 25 s at 62 °C. Relative gene copy number or expression level was obtained with the formula for fold induction, 2^−△△CT^. The C_T_ (threshold cycle) value represents the PCR cycle at which the *EPSPS* copy number or expression level passes the fixed threshold. Two experiments on three independent plant materials were performed to confirm the results, and each time point was repeated three times.

### Chimeric vector construction, plant transformation and overexpression of the *EPSPS* gene in *A*. *thaliana*

Total RNA was isolated, and cDNA was synthesized. The coding regions of *EPSPS* of *C*. *hederacea* and *C*. *arvensis* were amplified using the EPS-1f and EPS-1r primer pair (Table 1), and the complete *EPSPS* gene was inserted into the pMD19-T. The vector was verified by sequencing and then digested using *Xba*I/*Sma*I. The resulting product was cloned into the pBI121 vector, and the *35S*::*EPSPS* construct was obtained.

The expression vectors *35S*::*CaEPSPS* and *35S*::*ChEPSPS* were introduced into GV3101 *Agrobacterium tumefaciens*. The transformed *A*. *tumefaciens* were used to infect *A*. *thaliana* by the floral-dipping method^41^. T_1_ seeds were collected and grown under sterile conditions on media containing half-strength MS basal salt mixture, 1% sucrose and 40 mg. L^−1^ kanamycin. The surviving T_2_ seedlings showed a ratio of 3:1 KanR/KanS and were selected to produce T_3_ seeds. T_3_ lines containing the *EPSPS* gene were considered homozygous and used for further analysis. Three lines of each transgenic *A*. *thaliana* were used for glyphosate dose response analysis or GUS activity assay. Wild-type (WT) *A*. *thaliana* was used as a control.

To investigate the role of EPSPS in glyphosate, the seeds of transgenic *EPSPS* and WT *A*. *thaliana* were planted on plates containing half-strength MS salts and glyphosate (1.0 mM), respectively. The subsequent growth of these plants was assessed visually and photographed at 14 d after seeding.

### Quantitative analysis of GUS activity

To further investigate the *EPSPS* expression pattern, the *EPSPS* promoters from the two species were amplified using specific primers (ChEPS-Pf × ChEPS-Pr and CaEPS-Pf × CaEPS-Pr) (Table 1). The sequencing-verified promoters were isolated from pMD19-T using *Hind*III/*Xba*I digestion and then inserted into the pBI121 vector to generate *EPSPS-P*::*GUS*. The recombinant vectors were then verified by restriction digest. Expression vectors of *ChEPSPS-P*::*GUS* and *CaEPSPS-P*::*GUS* were finally introduced into *A*. *thaliana*. The method of plant transformation was described as above.

The GUS activity assay in transgenic *A*. *thaliana* seedlings used the methods described by Huang^42^. The data represent the means ± SD of triplicate measurements.

### Statistical analysis

Nonlinear regression analysis and ANOVA were used to determine dose–response curves for each species. The data were expressed as a percentage of dry weight compared to untreated control plants. Data from two repeated experiments with similar results were pooled. The GR_50_ was estimated by nonlinear regression using the logistic curve model:

$${\rm{Y}}={\rm{a}}/1+{{\rm{e}}}^{-({\rm{X}}-{{\rm{GR}}}_{50})/{\rm{b}}}.$$

In this equation, *a* is the difference between the upper and lower response limits, *GR*_50_ is the glyphosate dose that results in a 50% growth reduction, and *b* is the slope of the curve around *GR*_50_. The estimates were obtained using SigmaPlot software (version 12.0), and Tukey’s multiple range tests were used for comparation.

Data from the EPSPS copy number analysis and other experiment results were subjected to ANOVA, and the means were compared using Student’s *t-tes*t or Tukey’s multiple range tests. Means with different letters are significantly different at P = 0.05. All statistical analyses were performed using SPSS software (SPSS 17.0, SPSS Institute Inc.).

## Results

### Whole-plant bioassay

**Figure 1:**
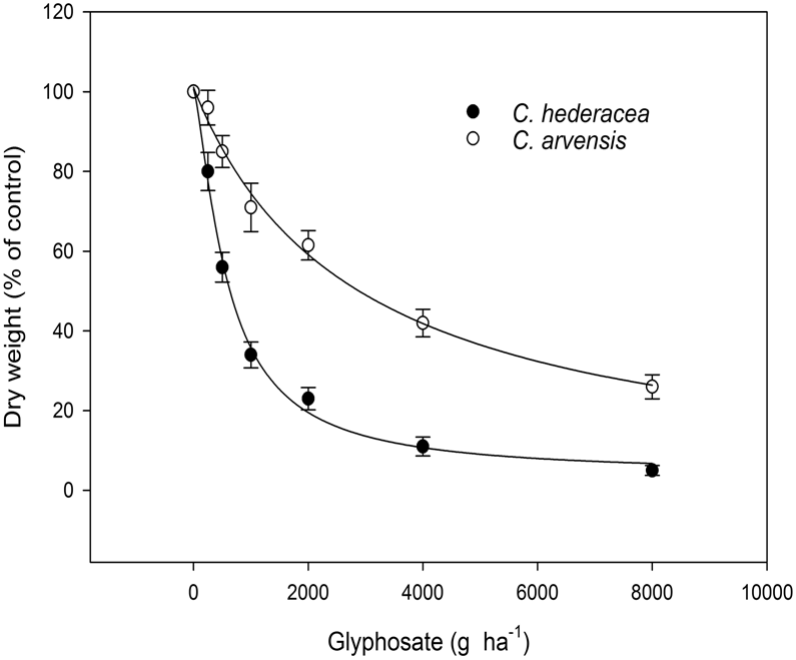
Dose–response assay of *C*. *hederacea* and *C*. *arvensis* treated with different glyphosate doses. Dry weight was expressed as a percentage of the untreated control. Each data point represents the mean ± SE of twice-repeated experiments containing three replicates each, and vertical bars represent the standard error.

The responses of *C*. *hederacea* and *C*. *arvensis* to glyphosate were different (Fig. 1). At the glyphosate field rate (1000 g ha^−1^), the growth of *C*. *hederacea* was reduced by approximately 70%, whereas the growth of *C*. *arvensis* was reduced by nearly 30%. The *C*. *arvensis* plants were not completely controlled by a glyphosate rate of up to 4000 g ha^−1^. The GR_50_ values for *C*. *hederacea* and *C*. *arvensis* were 562.1 and 2,866.3 g ha^−1^, respectively, and the calculated tolerant index was 5.1.

### Shikimic acid accumulation

**Figure 2:**
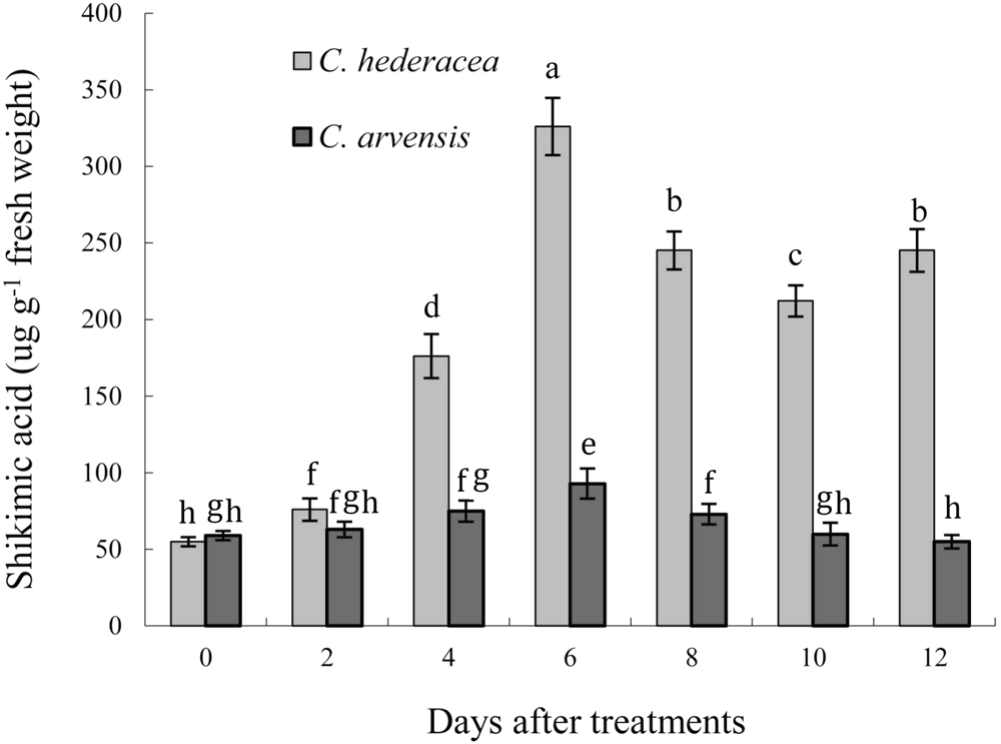
Shikimic acid accumulation in *C*. *hederacea* and *C*. *arvensis* after glyphosate treatments. Each data point represents the mean of twice-repeated experiments containing three replicates each, and vertical bars represent standard errors of the means. Means with different letters are significantly different at P = 0.05.

Basal shikimate acid levels were similar (55.1–59.2 µg g^−1^ FW) for *C*. *hederacea* and *C*. *arvensis* in our study. Shikimic acid accumulation exceeded the initial levels of untreated plants after glyphosate application (1000 g ha^−1^), and both species accumulated shikimate acid until 6 DAT. However, the two species thereafter differed in shikimate accumulation at 6 DAT, accumulation decreased in *C*. *arvensis* but fluctuated in *C*. *hederacea* (Fig. 2). Shikimic acid accumulation in *C*. *hederacea* (with a peak of 326.2 µg g^−1^ FW at 6 DAT) was 3.5 times higher than that in *C*. *arvensis* at 6 DAT.

### Sequence analysis of *EPSPS*

**Figure 3:**
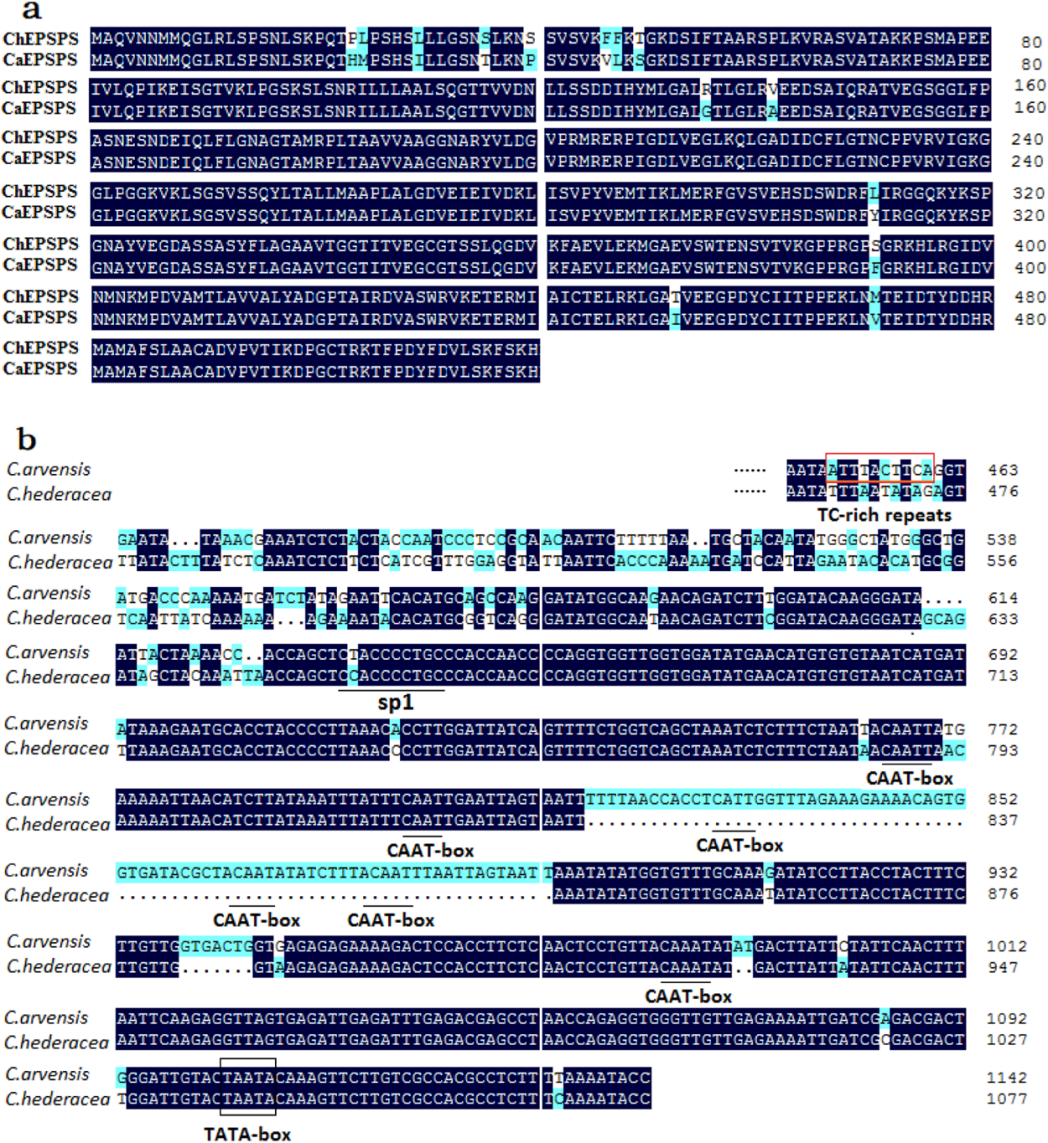
(**a**) Comparison of amino acid sequences of EPSPS from *C*. *hederacea* and *C*. *arvensis*. (**b**) Partial nucleotide sequences of the *EPSPS* promoters from *C*. *hederacea* and *C*. *arvensis*. TATA-box, CAAT-boxes and putative *cis*-acting elements were boxed or labeled.

Full-length *EPSPS* cDNAs were isolated from *C*. *hederacea* and *C*. *arvensis* (*ChEPSPS*, EU526078; *CaEPSPS*, EU698030) using specific primers. Sequence analysis revealed that both *ChEPSPS* and *CaEPSPS* consisted of a 1,563 bp open reading frame (ORF) encoding a polypeptide of 520 amino acids. The deduced amino acid sequences shared high similarity (identity was 97.31%). There are 14 different amino acids in EPSPS between the two species, and 6 sites were conserved (Fig. 3a). However, there were no point mutations, such as those mainly found at positions 102 or 106 in EPSPS, which have previously been associated with glyphosate resistance.

Fragments of 1,077 bp and 1,142 bp upstream of the *ChEPSPS* and *CaEPSPS* genes, respectively, were obtained by genome walking and designated as promoter regions (named *ChEPSPS-P* and *CaEPSPS-P*, respectively). PlantCARE analysis of *ChEPSPS-P* showed that a TATA box at −40 to −36 and three CAAT boxes at −350 to −152 were included in the promoter. Furthermore, a putative *cis*-acting sp1 element was found within the promoter sequence (Fig. 3b). Sequence analysis of *CaEPSPS-P* with PlantCARE showed the presence of common core promoter elements, including a “TATA-box” (−40 to −36), six “CAAT-box” (−379 to −156) and many *cis*-acting elements, such as spl, ARE, and GATA motifs. Furthermore, there was a *cis*-acting TC-rich repeat element, which is involved in defence and stress responsiveness, located in *CaEPSPS-P* (Fig. 3b).

### Response to glyphosate in transgenic *A*. *thaliana*

**Figure 4:**
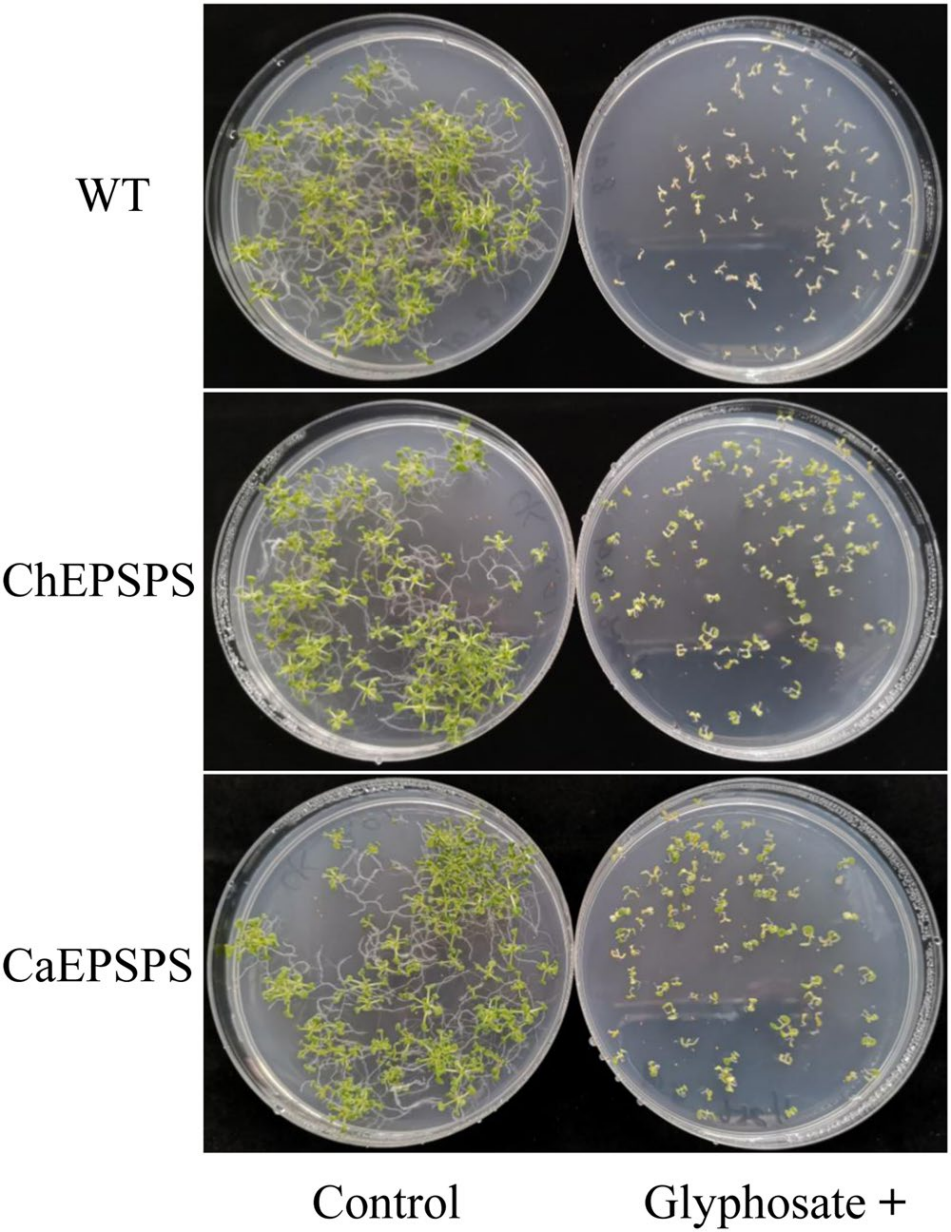
Comparison of glyphosate tolerance in WT, *ChEPSPS*, and *CaEPSPS* transgenic *A*. *thaliana*. Transgenic *EPSPS* and WT *A*. *thaliana* grown in half-strength MS solid medium either containing glyphosate (1.0 mM) or blank were photographed 14 d after seeding.

To investigate the role of *CaEPSPS* and *ChEPSPS* in response to glyphosate, three independent transgenic *A*. *thaliana* lines expressing either *EPSPS* gene and WT were assayed. Because the three *CaEPSPS or ChEPSPS* transgenic *A*. *thaliana* lines showed similar tolerance to glyphosate (data not shown), one line of *CaEPSPS* or *ChEPSPS* transgenic *A*. *thaliana* was selected for imaging. Based on Fig. 4, there was no obvious difference in plant growth among the WT, *CaEPSPS* and *ChEPSPS* transgenic *A*. *thaliana* in the absence of glyphosate. However, in the presence of glyphosate (1 mg L^−1^), the WT growth was inhibited, and the cotyledons turned yellow and died. In contrast, the *CaEPSPS* and *ChEPSPS* transgenic *A*. *thaliana* produced normal plants on Petri dishes and showed similar growth. Thus, the *CaEPSPS* and *ChEPSPS* genes similarly conferred the ability to withstand higher glyphosate treatments in transgenic *A*. *thaliana*. These results indicate that the amino acid differences in EPSPS were not the cause of glyphosate tolerance in *C*. *arvensis*.

### Comparison of *EPSPS* gene copy number and expression level

**Figure 5:**
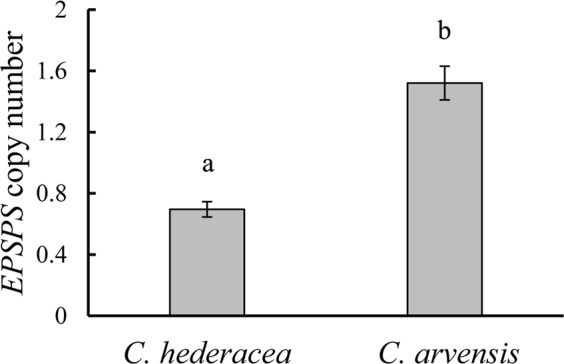
The *EPSPS* copy number detected in *C*. *hederacea* and *C*. *arvensis*. Values are mean ± SD, where n = 3 independent plants. Means with different letters are significantly different at P = 0.05.

As we found that the amino acid differences did not account for glyphosate tolerance in *C*. *arvensis*, the *EPSPS* gene copy number in both species was evaluated by qPCR using *GAPDH* as a normalization gene. The *EPSPS* copy number in the glyphosate-susceptible *C*. *hederacea* ranged from 0.64 to 0.75; however, the glyphosate-tolerant *C*. *arvensis* had higher relative *EPSPS* copy numbers, varying from 1.41 to 1.63 (Fig. 5), showing approximately 2 times higher copy number expression than that of *C*. *hederacea*. A higher *EPSPS* copy number indicated that *C*. *arvensis* could produce adequate EPSPS to bind glyphosate, thus conferring higher tolerance compared to *C*. *hederacea*.

**Figure 6:**
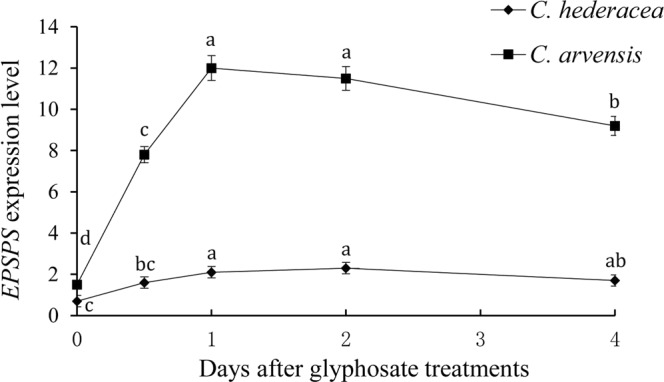
The *EPSPS* expression level detected at different times after glyphosate treatment. Data bars represent the mean ± SD of triplicate measurements. Means with different letters are significantly different at P = 0.05.

To examine the expression level of the *EPSPS* transcript in *C*. *arvensis* and *C*. *hederacea*, we carried out qPCR analysis with template cDNA derived from plants induced by 1000 g ha^−1^ glyphosate for different times. As shown in Fig. 6, glyphosate treatment induced a remarkable and steady increase of *EPSPS* expression in *C*. *arvensis* from 0.5 to 1 DAT with nearly 12 times higher peak induction than that of the untreated control, and then the *EPSPS* transcript level declined. In comparison, glyphosate caused a longer but weaker induction of *EPSPS* in *C*. *hederacea*. The induction began at 0.5 DAT and declined at 2 DAT. The peak induction in *C*. *hederacea* was much lower than that in *C*. *arvensis* (Fig. 6).

### *GUS* expression from the *EPSPS* promoter

**Figure 7:**
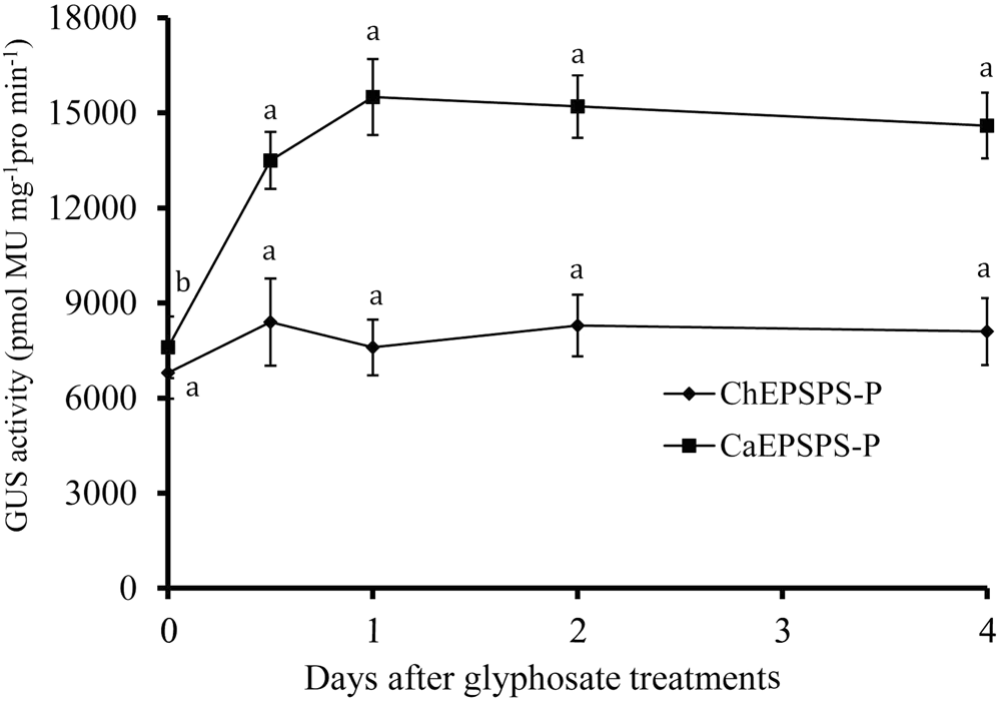
GUS activity detected at different times after glyphosate treatment. Data bars represent the mean ± SD of triplicate measurements. Means with different letters are significantly different at P = 0.05.

As the expression levels of the *EPSPS* genes induced by glyphosate in *C*. *arvensis* and *C*. *hederacea* were obviously different (Fig. 6), we assumed that the specific promoter was likely associated with the differences in *EPSPS* expression. Hence, we fused the *EPSPS* promoters to the *GUS* gene and transformed the recombinant vectors into *A*. *thaliana* plants to further investigate the *EPSPS* expression regulatory mechanism. The GUS activity in three transgenic *A*. *thaliana* lines expressing *ChEPSPS-P* or *CaEPSPS-P* was examined at 0.5, 1, 2 and 4 days after glyphosate application. The results showed that there was no significant difference of the GUS activity in the *ChEPSPS-P* transgenic *A*. *thaliana* throughout the experiment. In contrast, the GUS activity of the *CaEPSPS-P* transgenic *A*. *thaliana* was induced at much higher levels by glyphosate from 0.5 to 1 days. The peak induction was detected at 1 day after glyphosate application (Fig. 7). These results indicated that some *cis*-elements likely exist in *CaEPSPS-P* that are induced by glyphosate and drive GUS overexpression. This result was consistent with our hypothesis that the overexpression of *EPSPS* after glyphosate treatment in *C*. *arvensis* was likely mediated by a specific *EPSPS* promoter.

## Discussion

Several weeds, including *C*. *arvensis*, have been identified with different glyphosate tolerance levels^43–47^. The recommended glyphosate field doses are commonly 900 to 1500 g ha^−1^, although these doses vary according to the agronomic management and product marketing of the crops. Thus, *C*. *arvensis* (at GR_50_ level) is tolerant to glyphosate at 1.9–3.2 times the field dose and 5.1 times the level of the glyphosate-susceptible *C*. *hederacea* (Fig. 1). To achieve the complete control of *C*. *arvensis*, at least double the GR_50_ rate of glyphosate should be applied; however, this application rate will increase the selection pressure and accelerate the resistance evolution to glyphosate.

Glyphosate affects aromatic amino acid biosynthesis by inhibiting EPSPS, which is a critical enzyme in the shikimate pathway. Previous studies have employed shikimic acid accumulation as a parameter for discriminating glyphosate resistance^48–50^. For example, the inhibition of EPSPS by glyphosate in susceptible weeds usually results in shikimic acid accumulation. Furthermore, glyphosate-tolerant or glyphosate-resistant weeds accumulate shikimate at much lower levels than susceptible plants^51^. In our study, growth setback and eventual death were observed in *C*. *hederacea* owing to shikimic acid accumulation. This effect was due to the complete binding of EPSPS by glyphosate in *C*. *hederacea*, resulting in the accumulation of shikimic acid, whereas EPSPS in *C*. *arvensis* was not fully inhibited and would still maintain the shikimic pathway, thus leading to normal growth with slight developmental anomalies, such as deformed leaves and shortened internodes. Shikimate accumulation assays indicated that the glyphosate targeting of EPSPS plays a critical role in glyphosate tolerance in *C*. *arvensis*. Therefore, EPSPS alteration (mutation or amplification) is likely the major mechanism underlying glyphosate tolerance in *C*. *arvensis*.

*EPSPS* point mutations have been well established as major mechanisms of glyphosate resistance^26^. Some weeds displaying glyphosate resistance have a site mutation (particularly at the Pro106 codon) in the *EPSPS* gene^26^. Recently, *E*. *indica*^16,17^ and *Bidens Pilosa*^18^ with a double mutation reported as TIPS (Thr-102-Ile + Pro-106-Ser), have been found to have a high degree of glyphosate resistance. Three amino acid residues (Asp-71-Met, Ala-112-Ile, and Val-201-Met) and a 91Glu deletion in *EPSPS* were reported to be associated with natural tolerance to glyphosate in three lilyturf species^25^. In our study, six different amino acid substitutions were discovered in EPSPS in *C*. *arvensis*. To investigate the response of different EPSPS proteins to glyphosate, *EPSPS* genes were inserted into *A*. *thaliana*. Glyphosate response assays showed that the two transgenic *A*. *thaliana* shared similar glyphosate tolerance levels (Fig. 4). Therefore, target-site mutations are unlikely to account for glyphosate tolerance in *C*. *arvensis*.

To examine the possibility of *EPSPS* overexpression contributing to glyphosate tolerance in *C*. *arvensis*, both the basal and induced *EPSPS* mRNA levels were determined for the two species in this study. The *EPSPS* copy number for *C*. *arvensis* was 2 times higher than that of *C*. *hederacea* (Fig. 5). This result alone is not sufficient to explain the tolerance of *C*. *arvensis* at the whole plant level. However, the glyphosate-induced expression of the *EPSPS* gene in *C*. *arvensis* was highly enhanced after treatment compared to that in *C*. *hederacea* (Fig. 6). Multiple *EPSPS* copy numbers and/or increased expression of *EPSPS* have also been reported in other weed species, such as *D*. *chinensis*^24^, *O*. *japonicus*^25^, *A*. *palmeri*^52–54^, and *Conyza* species^55,56^. Therefore, a higher *EPSPS* copy number together with increased *EPSPS* expression likely play an important role in glyphosate tolerance in *C*. *arvensis*.

Gene expression is mostly regulated by the promoter. Different promoter regions may have distinctive regulatory functions^57^. In our study, there was a 77 bp extension in the *EPSPS* promoter of *C*. *arvensis*, which includes three CAAT-boxes. CAAT boxes are known to play important roles in enhancing the transcriptional level of the gene. Moreover, TC-rich repeats, which are involved in defence and stress responsiveness, are located in *CaEPSPS-P* (Fig. 3b). Thus, *cis*-elements, such as CAAT-boxes or TC-rich repeats, are likely induced by glyphosate treatment and improve the capacity to respond to glyphosate treatment via feedback regulation. In combination with *EPSPS* gene amplification, the *EPSPS* promoter containing specific *cis*-elements or increased transcription factor activity may increase EPSPS expression and confer glyphosate tolerance in *C*. *arvensis*. Further study will be necessary to detect the function of these *cis*-elements in the glyphosate feedback regulatory mechanism.

## Conclusion

We have demonstrated that *C*. *arvensis* is naturally tolerant to glyphosate at a much higher dose than glyphosate-susceptible *C*. *hederacea*. *C*. *arvensis* accumulated less shikimic acids when treated with glyphosate. The EPSPS of *C*. *arvensis* shares high similarity with that of *C*. *hederacea*, with six different conserved amino acids; however, the response to glyphosate in *EPSPS* transgenic *Arabidopsis* assays showed that these plants shared similar glyphosate tolerance levels. We also observed that the *EPSPS* copy number in *C*. *arvensis* was approximately 2 times higher than that of *C*. *hederacea*, and the *EPSPS* mRNA in *C*. *arvensis* could be highly induced by glyphosate. We conclude that the underlying basis for the glyphosate tolerance of *C*. *arvensis* is primarily due to high EPSPS gene copy numbers and specific promoter-mediated *EPSPS* overexpression after glyphosate treatment. This study could be of increased importance in weed management if the weeds share a similar glyphosate tolerance mechanism. Our future studies will focus on identifying the putative *cis*-elements of *CaEPSPS-P* in the glyphosate feedback regulatory mechanism.
